# Supplementary material for: Ultrahigh-Throughput Screening of High-β-Xylosidase-Producing Penicillium piceum and Investigation of the Novel β-Xylosidase Characteristics
Source: J Fungi (Basel). 2022 Mar 22;8(4):325. doi: 10.3390/jof8040325 (PMC9024563; doi:10.3390/jof8040325)
Supplement: Supplementary file 1 [file jof-08-00325-s001.zip › jof-1606869-supplementary.pdf]

A B

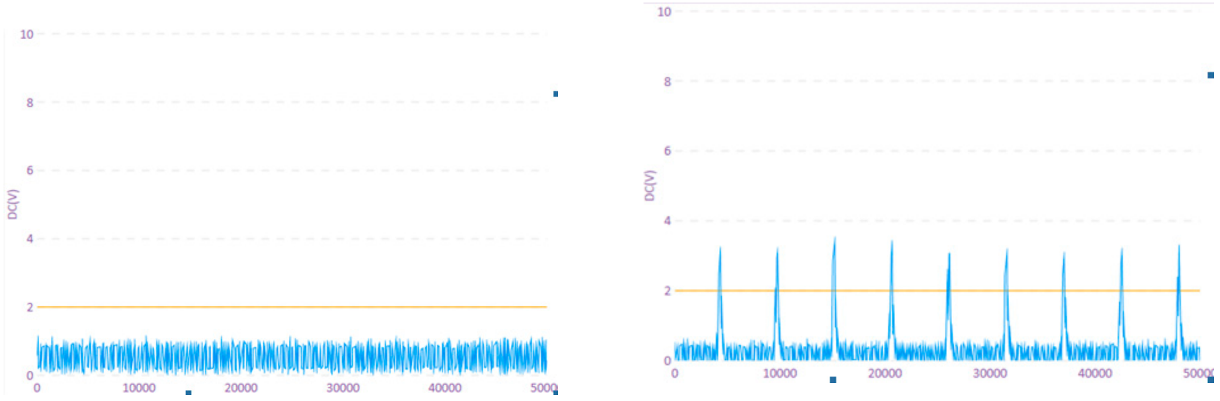

Figure S1: Comparison of fluorescence signal released from negative (with no β-xylosidase activity) and positive control (with high β-xylosidase activity).

MTLSVLTVASLVATALGSKVSVVVDASSKLGPWTPIHQYYGCDEPNYAYYPHGSAL
LKELGSLGKAQTYFRTHNLLTTGQPGLVGVPGLKWGSTNAYTLDENGMPVYNFTIV
DEIFDHYLANNVKPFLEIGFMPEALAVDPDPYFFDFDPAAGPDNIYTGWTHPPKSYER
WGKLIYEFTKHLVDRYGAKEVNQWPFEVWNEPNIPYWNGTTAEYYKLYDYTVRSV
LEALPTAHIGGPAVAGGASGSYLGDFLEHCSQGTNYATGEKGVPLDLISFHAKGVPQ
FINTTDTPGSSGYLQMDMSPQLQQIDEAFEVITSFHQYKQKPVFMSEYDPDGCAACTS
AAYDYRNGLMYGAYSAASFARAIDLAANRSVNLQAALTWAFEYEKNAILPNETGYF
DGFRVLSTQGIDKPVLNFHRMWSMLSGDRIKAESSAQIPIQEVLSNGIHGAQTDVGSL
ATLTEDGRALYVFLWHYHDNDLSFPDAQVSIDIEGLPAHFAEAKLTHYRVDNKHSNS
YAKWLSMGSPQSPTAEQYNELVAAGKLTTLGSPTSMHAHKGRLSTDLSLPIQALSLL
VFTAL

Figure S2: Amino acid sequence of PpBXL.The six identified peptides are marked with underline.
